# Conversational Agents to Support Pain Management: A Scoping Review

**DOI:** 10.1002/ejp.70016

**Published:** 2025-04-01

**Authors:** Filipe L. Souza, Hannah Bowman, Francis Yang, Negin Hesam‐Shariati, Jackson Linke, Yannick L. Gilanyi, Matthew D. Jones, Rafael Z‐Pinto, James H. McAuley, Rodrigo R. N. Rizzo

**Affiliations:** ^1^ School of Health Sciences, University of Vale Do Itajaí Itajaí Brazil; ^2^ Centre for Pain IMPACT, Neuroscience Research Australia Sydney New South Wales Australia; ^3^ School of Health Sciences, Faculty of Medicine University of New South Wales Sydney New South Wales Australia; ^4^ School of Psychology University of new South Wales Sydney New South Wales Australia

**Keywords:** artificial intelligence, artificial intelligence assistants, chatbot, conversational agents, pain management, review

## Abstract

**Background:**

Pain‐related conditions are the leading cause of years lived with disability globally. Managing pain presents significant challenges, including the need to address multiple biopsychosocial factors and the difficulty in delivering evidence‐based treatments. Digital health technologies, such as conversational agents, offer the potential for personalised and accessible pain management. However, the characteristics and effectiveness of these interventions are not yet fully understood. This scoping review aims to comprehensively evaluate the applications and effectiveness of conversational agents in supporting pain management in adults (i.e., healthy individuals at risk of developing pain, individuals currently experiencing pain and healthcare providers or students involved in managing pain conditions).

**Methods:**

Searches were systematically conducted across six databases—MEDLINE PubMed, ACM Digital Library, CINAHL, Embase, PsycINFO, Cochrane CENTRAL—and five trial registries from inception.

**Results:**

Twenty‐eight studies were included, focusing on capturing health information (*n* = 8), providing emotional support (*n* = 7), facilitating adherence to self‐management exercises (*n* = 6), delivering psychological treatment (*n* = 5), offering organisational support (*n* = 1) and educating healthcare providers (*n* = 1). These studies addressed conditions with pain as a central or common symptom, including dementia (*n* = 7), cancer (*n* = 5) and musculoskeletal disorders (*n* = 4), among others. None of the conversational agents on the market covered all four stages recommended for translational research (development, feasibility, effectiveness and implementation).

**Conclusion:**

The use of conversational agents in pain management is relatively new and involves diverse and promising appllications. However, evidence supporting their effectiveness in improving pain‐related outcomes remains limited and heterogeneous. Future reseacrh should prioritise feasibility, reliability, and user experience studies to inform the design of robust randomised controlled trials.

**Significance:**

This scoping review comprehensively examines the use of conversational agents (CAs) in adult pain management. The study identified six applications of CAs to support pain management and highlights a lack of high‐quality randomised controlled trials, particularly those preceded by development and feasibility studies. Clinicians and researchers can use these insights to guide future studies and improve applications of CAs in pain management.

## 1 Background

Pain is the most common reason for seeking care (Haas et al. 2023; Khatami et al. 2022). Approximately 30% of the world population reported moderate or severe pain in the last 30 days (Zimmer et al. 2022), and many report pain that persists for 3 months or more (i.e., chronic pain) (Mills et al. 2019). Pain‐related conditions, including headaches and musculoskeletal disorders (e.g., back pain, neck pain, knee pain), are the leading causes of years lived with disability worldwide (Vos et al. 2020). Chronic pain is often associated with multiple factors, including psychological distress, depression, anxiety, sleep problems, activity limitation, social participation restrictions (Cohen et al. 2021) and uncertainties regarding diagnosis, prognosis and treatment options (Lim et al. 2019).

Digital health solutions have been proposed to improve healthcare quality by facilitating data collection for consultation, providing timely information on the most beneficial treatment options, and delivering cost‐effective interventions (WHO. 2018). The use of conversational agents (CAs) in digital health solutions has become increasingly popular. A CA can be defined as a computer dialogue system that communicates with a human (Allouch et al. 2021). With recent technological advancements, CAs commonly use artificial intelligence (AI), including natural language processing and generative AI, such as ChatGPT (Xue et al. 2023), which may foster meaningful communication between users and machines (Xue et al. 2023). Despite being underutilised, CAs emerge as a highly customisable element in healthcare for diverse populations, with largely unexplored potential beyond daily health reports, feedback, alerts and patient recommendations (Kocaballi et al. 2019). Regarding chronic conditions, CAs have been used to deliver patient education, provide cognitive therapy protocols and offer emotional support in coping with chronic conditions (Uetova et al. 2024).

Reviews have demonstrated that CAs are used in healthcare for various purposes, such as gathering health information, sending medication and appointment reminders, delivering information (Dingler et al. 2021), providing mental health support, promoting physical activity and influencing health behaviours (Xue et al. 2023). A systematic review with meta‐analysis showed that AI‐based CAs may be beneficial in reducing symptoms of distress and depression (Li et al. 2023). However, the applications and effectiveness of CAs in supporting pain management have not yet been investigated. This scoping review aims to comprehensively evaluate the applications and effectiveness of CAs to support pain management in adults.

## 2 Methods

### 2.1 Protocol Registration and Design

We conducted a scoping review to investigate the current applications and effectiveness of CAs for supporting pain management in adults. We reported the scoping review according to the Preferred Reporting Items for Systematic Reviews and Meta‐Analyses for Scoping Review (PRISMA‐ScR) reporting guidelines (Chen et al. 2023). We did not formulate a hypothesis to investigate the applications of CAs for supporting pain management because it is a scoping review (Tricco et al. 2016). However, since we expected to include randomised controlled trials (RCTs), we formulated a specific hypothesis regarding the effectiveness of CAs in improving pain management (e.g., delivery of treatments, knowledge of health professionals) or outcomes. We hypothesised that CAs improve pain management and health outcomes compared to control groups. The protocol was prospectively registered on the Open Science Framework (OSF; https://osf.io/23p7g). We updated the protocol after the screening to provide details on how we plan to analyse and report the findings and quality of RCTs, following the significant inclusion of these trials in the scoping review.

### 2.2 Selection Criteria and Search Strategy

#### 2.2.1 Population

We included studies with participants aged 18 years and older that recruited healthy individuals at risk of developing pain (e.g., healthy workers with physical demand at risk of developing back pain), individuals currently experiencing pain or health providers or students managing pain conditions.

#### 2.2.2 Intervention

The inclusion criteria were intentionally designed to comprehensively map the literature on CAs, reflecting the broader approach characteristic of scoping reviews (Tricco et al. 2016). We included studies investigating the role of CAs in supporting pain management, which is defined as a coordinated and interdisciplinary approach that might involve consumers (e.g., patients), health providers and/or the healthcare system to alleviate suffering or improve the quality of life for individuals experiencing pain (Medicine, 2012). We defined a CA as an independent dialogue system or computer program that can communicate (verbally or non‐verbally) with a human. These agents had to process inputs from speech, text, video or other sensors (Allouch et al. 2021).

We included studies investigating CAs to understand the input provided by the user in a conversation style and deliver appropriate information, advice, feedback or actions, which can be conveyed via text, speech or by controlling a physical or virtual entity. There were no restrictions regarding length, frequency, technology or cointerventions. The CA had to be intended to manage pain, including but not limited to assessment, prevention, treatment and education.

#### 2.2.3 Outcomes

We included pain‐related outcomes (e.g., pain intensity, pain interference), physical function, psychological outcomes (e.g., anxiety, depression) and user experience outcomes (e.g., engagement, usefulness).

#### 2.2.4 Study Design

We included all types of studies, including RCTs, pilot trials and observational studies (e.g., feasibility, qualitative) that investigated the role of CAs in pain management. We also included protocols for ongoing RCTs and published abstracts to provide a comprehensive overview of current research activity and identify emerging trends in the field. Including these sources is consistent with the purpose of scoping reviews, which aim to broadly map the literature and inform future systematic reviews or trials. This inclusive approach ensures a thorough understanding of the available evidence, even when outcome data still need to be reported, to facilitate the identification of potential gaps and areas for future research (Gottlieb et al. 2021). There were no restrictions regarding language or date of publication.

#### 2.2.5 Exclusion Criteria

Studies were excluded if they focused on CAs unrelated to pain management (e.g., targeting general mental health or other non‐pain‐related domains), lacked details about the intervention or its functionality, included participants younger than 18 or were duplicate publications without unique data.

#### 2.2.6 Search Strategy

We searched six databases—MEDLINE PubMed, ACM Digital Library, CINAHL, Embase, PsycINFO, Cochrane Central Register of Controlled Trials (CENTRAL) and five trial registries—EU Clinical Trials Register, ClinicalTrials.gov, International Clinical Trials Registry Platform (ICTRP) and Australian New Zealand Clinical Trials Registry (ANZCTR) and OSF from inception. We used terms related to ‘conversational agents’ and ‘pain’ categories, separated by ‘OR’ within each term or ‘AND’ for terms across different categories. The search strategy is detailed in Appendix S1. In addition to searching scientific databases, we emailed the corresponding authors of all included studies and trial registrations to inquire whether the CAs studied were already commercially available or still under investigation. To ensure comprehensive information about the commercialisation status of all analysed technologies, we also conducted supplementary searches on Google.

### 2.3 Data Management and Screening

We used Covidence (Veritas Health Innovation 2023) to remove duplicates and screen records. Two reviewers independently screened the titles and abstracts of all records and then the full text based on the eligibility criteria. Where multiple records of the same trial were present, preference was given to the most recent record and the record(s) that provided data for the review. In the case of disagreement, a third reviewer resolved the discrepancies during the screening process.

### 2.4 Data Extraction

Two authors (R.R.N.R. and F.Y.) developed and pilot‐tested a data extraction form, which was used by at least two independent reviewers (H.B., R.R.N.R., Y.G., J.L. and M.J.) to extract data from all eligible records. Discrepancies were resolved through discussion with a third reviewer when necessary. Data extraction focused on study characteristics, participant details, intervention and CA attributes and reported outcomes. A detailed description of the extracted variables, including classification criteria and rationale, is provided in Appendix S2.

### 2.5 Clinical Research Phase Studies

The complex intervention evaluation framework, recommended by the Medical Research Council, guides the assessment of interventions across stages: development, feasibility, effectiveness and implementation. Our review also considered the commercialisation stage for interventions already available for patient acquisition outside experimental settings (Regier et al. 2022). This framework acknowledges that, after initial evaluations (i.e., development and feasibility), further investigations (i.e., effectiveness, implementation, regulation) are necessary to ensure the healthcare intervention is safe, effective and implementable. Therefore, the present review organised the research output on CAs for pain management by the evaluation stage.

### 2.6 Missing Data

Where data was missing, we contacted study authors twice via email (1 week apart). Data were considered unobtainable if there was no reply within 1 week after the final email.

### 2.7 Risk of Bias and Certainty of Evidence

Two reviewers independently assessed the risk of bias in the RCTs using the Cochrane Risk‐of‐Bias‐2 (RoB2) tool (Sterne et al. 2019). This tool assesses several domains of bias: bias from randomisation, deviations from intended interventions, missing outcome data, outcome measurements and selective reporting. According to the RoB2 tool classifications, two reviewers judged the risk of bias as either low, some concerns or high.

As described in our preregistration, in the case of meta‐analysis, the certainty of evidence would be assessed using the Grading of Recommendations, Assessment, Development and Evaluations framework (GRADE, according to the protocol) (Brozek et al. 2021). Scoping reviews, including the possibility of meta‐analysis, aim to generate evidence‐based maps and hypotheses. Our hypothesis for such an analysis is that CAs are more effective than controls in managing pain, consistent with the scoping meta‐review approach (Sarrami‐Foroushani et al. 2015).

## 3 Results

**FIGURE 1:**
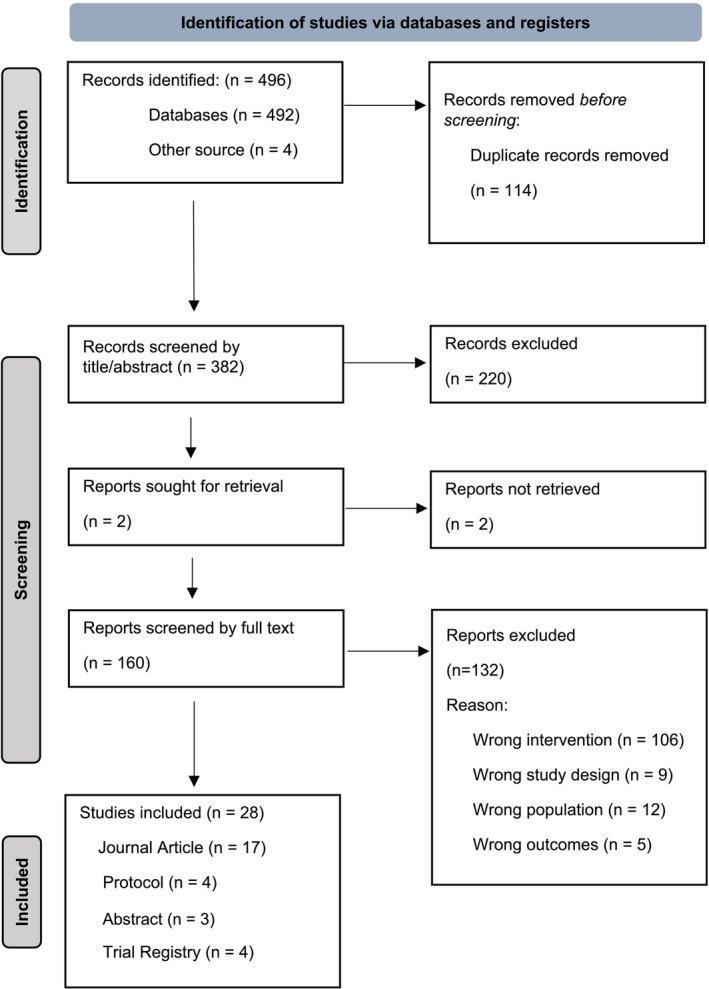
PRISMA flow diagram.

We retrieved 496 records from the search (Figure 1), including 492 from the databases and four from other sources (i.e., searching the references of the included studies). After removing 114 duplicates, we screened 382 titles and abstracts for inclusion. We excluded 222 records due to wrong intervention (*n* = 105), wrong study design (*n* = 9), wrong population (*n* = 12) and wrong outcomes (*n* = 5). We retrieved the full text of 160 potentially eligible records. We included 28 articles, of which 17 were journal articles, four protocols, four trial registers and three abstracts.

### 3.1 Study Characteristics

The 28 articles included in our scoping review were published between 2011 and 2023, with 78% (*n* = 22) published in the past five years. The studies originated from nine countries, predominantly from the United States of America (*n* = 10, 35%). Study characteristics are outlined in Table 1.

**TABLE 1:** Study characteristics.

First author, year	Study type	Population	Country	Study design	Sample size	Study aim
McDonald et al. (2011)	Journal Article	Osteoarthritic pain	USA	Parallel RCT	30	Pilot test a cost‐effective virtual pain communication coaching intervention for the effect on older adults' communication of their osteoarthritis pain and pain management needs
McDonald et al. (2012)	Journal Article	Osteoarthritic pain	USA	Parallel RCT	35	Pilot test the effects of a virtual pain coach on ambulatory Spanish‐speaking older adults with pain from osteoarthritis
Khumrin et al. (2017)	Journal Article	Abdominal conditions	Australia	Case Study	208	Describe the development of a machine‐learning model to support medical students' diagnostic decisions
Demange et al. (2019)	Abstract	Dementia with chronic pain	France	Crossover RCT	53	Evaluate effectiveness of a social robot as a distracting and pleasant stimulus, during painful care situations in patients living with dementia
Pu et al. (2019)	Journal Article	Dementia and chronic pain	Australia	Cross‐sectional	11	Explore how people with mild to moderate dementia and chronic pain perceive a social robot as an alternative intervention to manage their pain and mood
Hauser‐Ulrich et al. (2020)	Journal Article	Chronic pain	Switzerland	Parallel RCT	102	Evaluate the implementation and effectiveness of a CBT text‐based healthcare chatbot intervention for pain management
Junior et al. (2020)	Abstract	Cancer	Brazil	Case Series	107	Describe a chatbot with artificial intelligence developed to collect PROs and to optimise adherence to systemic cancer treatment
Pu et al. (2020)	Journal Article	Dementia and chronic pain	Australia	Parallel RCT	43	Test feasibility of collecting cortisol via salivary swab as an indicator of stress in people with dementia and chronic pain who used a social robot
Anan et al. (2021)	Journal Article	Neck/shoulder stiffness and low back pain	Japan	Parallel RCT	121	Evaluate improvements in musculoskeletal symptoms in workers with neck/shoulder pain/stiffness and low back pain after use of exercise‐based AI assisted interactive health promotion system
Hunt et al. (2021)	Journal Article	Irritable bowel syndrome	USA	Crossover RCT	121	To test the efficacy of a novel app, Zemedy, as a mobile digital therapeutic that delivers a comprehensive CBT program to individuals with IBS
Kowatsch et al. (2021)	Journal Article	MKD	Switzerland	Cross‐sectional	67	Assess the role of Hybrid Ubiquitous Coaching (HUC) in improving adherence to home exercises across four studies
Ma et al. (2021)	Journal Article	Head and neck cancer	USA	Cross‐sectional	84	To report the early experience of using an automated chatbot for patient‐reported outcomes and symptom self‐management in head and neck cancer patients undergoing radiation treatment
Pu et al. (2021)	Journal Article	Dementia and chronic pain	Australia	Parallel RCT	43	Investigate the effect of a social robot intervention on sleep and motor activity in nursing home residents living with dementia and chronic pain
Leo et al. (2022)	Journal Article	MKD	USA	Cohort	61	Assess the feasibility of introducing a digital mental health intervention in an outpatient orthopaedic setting to patients with coexisting symptoms of depression and/or anxiety
Pu et al. (2022)	Protocol	Dementia (chronic pain)	Australia	Parallel RCT	NR	Test feasibility of PainChek app to assess pain for people with dementia living in residential aged care facilities
Silva et al. (2022)	Protocol	Peripheral arterial disease	NR	Parallel RCT	NR	Evaluate effectiveness of a home‐based exercise therapy program prescribed by healthcare professionals as a treatment for PAD, along with a behavioural change and motivational intervention (WalkingPad app with virtual assistant) to promote exercise adherence
Sinha et al. (2022)	Journal Article	MKD	USA	Cohort	51	Evaluate user retention and engagement with an artificial intelligence‐led digital mental health app (Wysa for Chronic Pain) that is customised for individuals managing mental health symptoms and coexisting chronic pain
Truica et al. (2022)	Abstract	Cancer	USA	Parallel RCT	NR	Investigate the use of a computer tablet‐based supportive care program (Nurse AMIE) in symptom management among cancer survivors
Blasco et al. (2023)	Protocol	Knee osteoarthritis	Spain	Parallel RCT	NR	Determine effectiveness of a virtual assistant to promote adherence to home rehabilitation
Cavalieri et al. (2023)	Protocol	Head and neck cancer	Italy and UK	Parallel RCT	NR	Reduce, with non‐invasive tools, the proportion of head and neck cancer survivors experiencing a clinically relevant reduction in QoL during follow‐up
Cheng et al. (2023)	Journal Article	MKD	USA	Cross‐sectional	30	Identify behavioural mechanisms that may mediate changes in mental and physical health following use of a chatbot for chronic pain
Murali et al. (2023)	Journal Article	Acute pain	USA	Parallel RCT	9	Describe preliminary work on an Embodied Conversational Agents (ECA) that elicits individual pain descriptions, along with a pilot feasibility study
Pu et al. (2023)	Journal Article	Dementia (chronic pain)	Australia	Cross‐sectional	34	Explore the experiences of residents with dementia, family and formal carers with; (1) pain assessment and management for residents with dementia; (2) the use of the PainChek app for pain assessment; and (3) the use of a social robot for pain management in residents with dementia
Schmitz et al. (2023)	Journal Article	Metastatic breast cancer	NR	Crossover RCT	42	Test a virtual assistant for addressing symptoms in metastatic breast cancer patients using the Amazon Echo Show with Alexa
ACTRN12621001010886 (2021)	Trial Registry	Dementia (chronic pain)	Australia	Parallel RCT	NR	Evaluate the effectiveness and economic value of technologies (the PainChek app and PARO robotic seal) on pain management in residential aged care, particularly for people with dementia
NCT03157362 (2022)	Trial Registry	Chronic pain	USA	Crossover RCT	NR	Examine the effects of transportation via social presence in ‘near’ and ‘far’ virtual environments, on pain threshold in two groups: (1) healthy volunteers in an induced pain task and (2) older adults suffering from pain
NCT06070415 (2023)	Trial Registry	Low back pain	Spain	Parallel RCT	NR	Evaluate the adherence to home exercises in patients with low back pain
NCT06070441 (2024)	Trial Registry	Cervical pain	Spain	Parallel RCT	NR	Verify whether the use of a Chatbot as a means of communication can produce improvements in patient adherence and clinical results

Abbreviation: NR, not relevant or reported.

### 3.2 Study Design

We identified six categories of study designs. There were nine observational studies (comprising 5 cross‐sectional studies, 1 case series, 1 case study and 2 cohort studies), 9 RCTs, 3 RCT protocols, 4 RCT trial registries and 2 RCT abstracts (comprising 15 parallel and 4 crossover trials).

#### 3.2.1 Setting

Approximately half of the studies reported the setting where participants received the intervention (*n* = 16, 57%). The most common settings for investigating CAs in the studies were aged care facilities (*n* = 7, 25%) and outpatient settings (*n* = 6, 21%). Participants were primarily recruited through convenience sampling (*n* = 14, 50%) and local medical practices in which participants were already presenting for standard care (*n* = 7, 25%).

#### 3.2.2 Participant Characteristics

The 28 studies included 1252 participants in total. Most studies reported a sample size (*n* = 18, 68%). The median sample size was 65 participants, ranging from 1 to 208.

Sixteen (57%) studies reported age. The mean age of participants ranged from 32 (10.2 SD) to 86.48 (8.81 SD) years. Sixteen (57%) studies reported on sex distribution, with females comprising approximately 63% (*n* = 913) of these studies' total reported sample size. Seven (25%) studies reported on race, with the majority being White (75.3%). Only five (17%) studies reported on education, with one stating 43.1% of participants had a university degree, another reporting 57.1% had a 4‐year university degree or higher, and the last two reporting late primary school to early high school‐level education, one stating 22% of participants had at least 7 years of education.

We included studies investigating CAs in six conditions with pain as a central or common symptom, including pain and dementia (*n* = 7), cancer (*n* = 5), musculoskeletal conditions (*n* = 4), irritable bowel syndrome (*n* = 1), peripheral artery disease (*n* = 1) and abdominal conditions (*n* = 1).

The included studies, study types, population, country of the study, study objective and sample size are reported in Table 1.

### 3.3 Conversational Agents Characteristics

**FIGURE 2:**
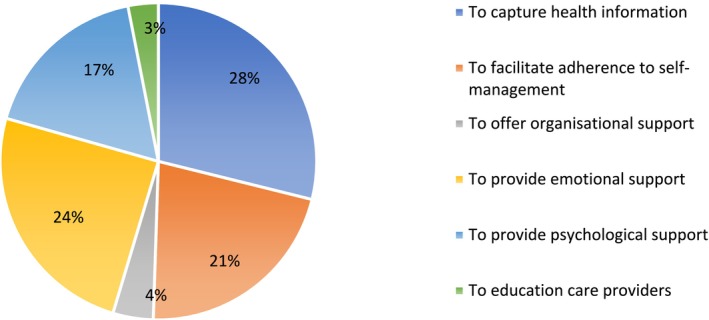
The purpose of the interventions used in the included studies.

We identified four types of CAs, the most common being chatbot design (*n* = 13) (Table 2). The others included therapeutic robotics (*n* = 7), which could communicate through body language; virtual reality/assistant/coach (*n* = 7), which refers to the use of an avatar or digital rendering of an individual; and computer‐aided learning system design (*n* = 1), which broadly refers to the use of integrative technology to assist user learning on a particular subject. We identified six intervention intentions describing the role of the CAs (Figure 2). The most common intention was to capture health information (*n* = 8), followed by providing emotional support (*n* = 7), facilitating adherence to self‐management physical exercises (*n* = 6), providing psychological treatment for pain management (*n* = 5), offering organisational support (*n* = 1) and educating health care providers (*n* = 1).

**TABLE 2:** Characteristics of conversational agents.

First author, year	Conversational agent intervention (control)	Conversational agent purpose (users)	Service provision (knowledge domain, constraint, type of service)	Technology (machine learning, natural language processing; generative AI)	User interface (input mode: text‐based; voice‐based; visual‐based or multimodal)	User interface (output mode: text‐based; voice‐based; visual‐based or multimodal)	Personalisation level	Security and privacy details	Duration (Frequency)
McDonald et al. (2011)	Virtual coach (pain education via video)	Assist older adults in practising pain communication with healthcare practitioners, promoting structured verbalisation and feedback (patients)	Closed, unconstrained, intrapersonal	Natural language processing	Voice‐based	Multimodal (voice and visual‐based)	Selection of personally relevant pain	NR	NR (NR)
McDonald et al. (2012)	Virtual coach (pain education via video)	Assist older adults in practising pain communication with healthcare practitioners, promoting structured verbalisation and feedback (patients)	Closed, unconstrained, intrapersonal	Natural language processing	Voice‐based	Multimodal (voice and visual‐based)	Selection of personally relevant pain	NR	1 month (NR)
Khumrin et al. (2017)	Computer‐aided learning system (NR)	Provide personalised diagnostic feedback to students using machine learning, comparing user decisions with predictive models (clinician)	Closed, constrained, interpersonal	Machine learning	Text‐based (patient records)	Text‐based	Assess students' performance	NR	NR (NR)
Demange et al. (2019)	Therapeutic robotic seal (usual care)	Provide distraction and comfort during painful care procedures for individuals with advanced dementia (patients)	Closed, constrained, intrapersonal	AI	Multimodal (audio, movement and visual‐based)	Multimodal (voice, tactile, light and movement‐based)	Behave as the patient prefers^a^	NR	3 weeks (NR)
Pu et al. (2019)	Therapeutic robotic seal (usual care)	Provide pain relief and emotional support for aged care residents with dementia through interactive social and sensory engagement (patients)	Closed, constrained, intrapersonal	AI	Multimodal (audio, movement and visual‐based)	Multimodal (voice, tactile, light and movement‐based)	Behave as the patient prefers^a^	NR	6 weeks (5 times a week)
Hauser‐Ulrich et al. (2020)	Chatbot (interaction with chatbot with no pain‐related content)	Support pain self‐management through psychoeducation and CBT‐based coping strategies via daily text messages (patients)	Closed, unconstrained, interpersonal	Generative AI	Multimodal (text, audio and figures‐based)	Text‐based	Communicating with a sense of humour and using emojis	NR	2 months (daily or other daily)
Junior et al. (2020)	Chatbot (NR)	Monitor symptoms and treatment adherence through active searches for adverse events, medication adherence and depression screening, plus patient‐initiated interactions (patients)	Closed, constrained, interpersonal	Generative AI	Text‐based	Text‐based	Respond to spontaneous patient demands	NR	NR (NR)
Pu et al. (2020)	Therapeutic robotic seal (usual care)	Provide pain relief and emotional support for aged care residents with dementia through interactive social and sensory engagement (patients)	Closed, unconstrained, intrapersonal	AI	Multimodal (audio, movement and visual‐based)	Multimodal (voice, tactile, light and movement‐based)	Behave as the patient prefers^a^	NR	6 weeks (5 times a week)
Anan et al. (2021)	Chatbot (usual care)	Motivate exercise and provide daily instructions to relieve musculoskeletal pain through text messages and motivational prompts (patients)	Closed, constrained, interpersonal	Generative AI	Text‐based	Multimodal (text and visual‐based)	Notification time could be changed by the users to a time convenient for them	NR	12 weeks (daily)
Hunt et al. (2021)	Chatbot (waitlist)	Support IBS management through CBT by guiding users through psychoeducation, relaxation, cognitive restructuring and behavioural exercises (patients)	Closed, constrained, interpersonal	Generative AI	Text‐based	Multimodal (text and visual‐based)	Patients could reach out to technical support if experienced technical difficulties	NR	8 weeks (self‐paced)
Kowatsch et al. (2021)	Chatbot (NR)	Support remote exercise adherence through reminders, motivation and psychoeducation via smartphone and augmented reality (patients)	Closed, constrained, interpersonal	Virtual reality	Multimodal (text and movement‐based)	Multimodal (text and visual‐based)	Reminders via WhatsApp messages	NR	4 weeks (3 times a week)
Ma et al. (2021)	Chatbot (NR)	Enable adverse event reporting and provide self‐care education through interactive scheduled and on‐demand chats with AI‐guided self‐reporting (patients)	Closed, constrained, interpersonal	Generative AI	Text‐based	Text‐based	Access unscheduled chats on demand	NR	4 weeks (weekly)
Pu et al. (2021)	Therapeutic robotic seal (usual care)	Provide pain relief and emotional support for aged care residents with dementia through interactive social and sensory engagement (patients)	Closed, unconstrained, intrapersonal	AI	Multimodal (audio, movement and visual‐based)	Multimodal (voice, tactile, light and movement‐based)	Behave as the patient prefers^a^	NR	6 weeks (5 times a week)
Leo et al. (2022)	Chatbot (NR)	Provide mental health and pain management support through AI‐driven chatbot and human coaching, delivering CBT, DBT, mindfulness and other therapeutic techniques (patients)	Closed, constrained, interpersonal and intrapersonal	Generative AI	Text‐based	Text‐based	Human ‘coaches’ with master's degrees in psychology	NR	8 weeks (daily)
Pu et al. (2022)	Therapeutic robotic seal (usual care)	Provide pain relief and emotional support for aged care residents with dementia through interactive social and sensory engagement (patients)	Closed, unconstrained, intrapersonal	AI	Multimodal (audio, movement and visual‐based)	Multimodal (voice, tactile, light and movement‐based)	Behave as the patient prefers^a^	Participant data de‐identified when disseminated	4 weeks (1–2 times a week)
Silva et al. (2022)	Virtual assistant (diary)	Monitor and promote adherence to a walking program through a virtual assistant that tracks progress and provides feedback (patients)	Closed, constrained, intrapersonal and interpersonal	Generative AI	Text‐based	Text‐based	Congratulatory messages were sent on each participant's birthday, as well as congratulatory messages for being part of the study every month	Storing, processing and protecting personal data will respect the Portuguese Law No. 58/2019 of 8 August	24 weeks (at least 3 times a week)
Sinha et al. (2022)	Chatbot (NR)	Provide emotional support and chronic pain management through daily check‐ins, mood tracking, CBT‐based interventions and guided meditations (patients)	Closed, constrained, interpersonal and intrapersonal	Generative AI	Text‐based	Text‐based	The effort is encouraged with a weekly report that offers insights and a new suite of tools.	NR	8 weeks (daily)
Truica et al. (2022)	Virtual assistant (access to supportive care materials)	Monitor symptoms and deliver evidence‐based supportive care through daily interactions with a nurse avatar, providing self‐care interventions. (patients)	Closed, constrained, intrapersonal and interpersonal	Generative AI	Text‐based	Multimodal (text, audio and visual‐based)^a^	NR	NR	NR (daily)
Blasco et al. (2023)	Chatbot (usual area and information brochure)	Supervise postoperative rehabilitation by providing motivational messages, reminders, training instructions and adherence monitoring (patients)	Open, unconstrained, intrapersonal	Generative AI	Text‐based	Text‐based	A physiotherapist contacts the patient by message or phone call if the patient's question does not have predefined answers in the Chatbot	NR	1 year (NR)
Cavalieri et al. (2023)	Chatbot (usual care)	Monitor quality of life and identify health‐related issues through an interactive chatbot that tracks symptoms and provides guidance (patients)	Closed, unconstrained, interpersonal	Generative AI	Multimodal (GPS, light, text and activity‐based)	Multimodal (text and visual‐based)	NR	The Data was transferred and stored in the central BD4QoL Repository hosted by Partner INETUM	24 months (NR)
Cheng et al. (2023)	Chatbot (NR)	Support mental health and chronic pain management through AI‐driven coaching, CBT techniques, mindfulness and guided sleep meditations (patients)	Closed, constrained, interpersonal and intrapersonal	Generative AI	Text‐based	Text‐based	Counsellors with master's degrees in psychology to deliver therapeutic content like: cognitive behavioural therapy, behavioural activation, motivational interviewing, etc.	All study data were stored in a secure electronic REDCap database	1 month (3 times a week)
Murali et al. (2023)	Virtual assistant (non‐empathic feedback)	Capture health and pain information through open‐ended prompts and provide an empathic summary using verbal and non‐verbal communication (patients)	Closed, unconstrained, interpersonal	Natural language processing	Multimodal (voice and gesture‐based)	Multimodal (voice and visual‐based)	NR	NR	1 h (once off)
Pu et al. (2023)	Therapeutic robotic seal (usual care)	Provide pain relief and emotional support for aged care residents with dementia through interactive social and sensory engagement (patients)	Closed constrained, intrapersonal	AI	Multimodal (audio, movement and visual‐based)	Multimodal (voice, tactile, light and movement‐based)	Behave as the patient prefers^a^	NR	3 weeks (5 times a week)
Schmitz et al. (2023)	Virtual assistant (usual care)	Monitor symptoms and provide supportive care for cancer patients through daily check‐ins, empathetic responses, intervention recommendations and clinician alerts (patients)	Closed, constrained, intrapersonal	Generative AI	Multimodal (voice and text‐based)	Multimodal (text, audio and visual‐based)^a^	NR	NR	6 months (self‐paced)
ACTRN12621001010886 (2021)	Therapeutic robotic seal (non‐interactive plush toy)	Provide emotional support and symptom relief for people with dementia through sensory interactions, expressive movements and adaptive responses (patients)	Closed, constrained, intrapersonal	AI	Multimodal (audio, movement and visual‐based)	Multimodal (voice, tactile, light and movement‐based)	Behave as the patient prefers^a^	NR	4 weeks (5 days a week)
NCT03157362 (2022)	Chatbot (home exercise, usual care and education materials)	Induce social presence through scripted avatar conversations in virtual environments during pain tasks (patients)	Closed, constrained, interpersonal	Generative AI	Multimodal (text and visual‐based)	NR	NR	NR	12 weeks (NR) and one week (3 times a week)
NCT06070415 (2023)	Home exercises with chatbot (home exercise without chatbot)	Promote adherence to home physiotherapy by providing instructional messages and videos on exercises, with guidance on repetitions and techniques (patients)	NA	Generative AI	Multimodal (text and visual‐based)	NR	NR	NR	12 weeks (NR)
NCT06070441 (2024)	Chatbot (usual care and education sessions)	Promote adherence to home physiotherapy for cervical injuries by providing instructional messages and videos on exercises, including repetition guidance (patients)	NA	Generative AI	Multimodal (text and visual‐based)	NR	NR	NR	12 weeks (NR)

*Note:* Natural language processing = Technologies that interpret and understand natural language. It involves analysing text and/or speech to process commands and generate responses.

Abbreviations: CBT, cognitive behavioural therapy; DBT, dialectical behavioural therapy; IBS, irritable bowel syndrome; NR, not reported or relevant.

^a^ It was necessary to research sources outside the included study to obtain information about the technology.

The knowledge domain of the CAs was mostly closed (*n* = 24, 85%) and constrained to specific pre‐programmed topics (*n* = 15, 53%). The service provided by the CAs was mostly for transmitting information without much intimate connection to users (i.e., interpersonal; *n* = 16, 57%). The goal of the CAs' interactions was largely conversational (*n* = 14, 50%) and informative (*n* = 11, 39%).

Most studies required communication with the CAs at least once a week (*n* = 15, 53%), including daily use (*n* = 5), weekly use (*n* = 3), five times a week (*n* = 5), at least three times a week (*n* = 2) and self‐paced (*n* = 2). The other studies did not report communication frequency, or the frequency was unclear.

### 3.4 Evaluation Stages of the Conversational Agents

**FIGURE 3:**
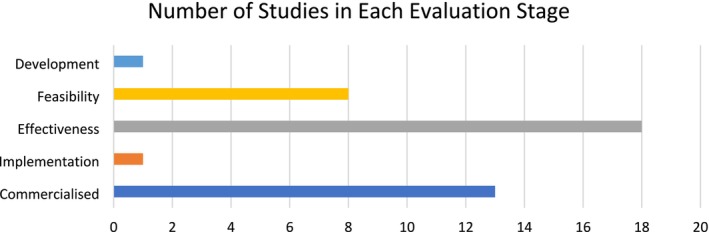
Number of studies in each evaluation stage of the lifecycle of healthcare interventions.

The complex intervention evaluation framework was used to evaluate the included studies (Appendix S2). We identified studies in the development (*n* = 1, < 1%), feasibility (*n* = 8, 28%), effectiveness (*n* = 18, 64%), implementation (*n* = 1, < 1%) and commercialisation (*n* = 13, 46%) stages (Figure 3).

Five CAs had been commercialised and featured in 13 of our included studies (46%). Of these, the marketed focus ranged from mental health (PARO; Wysa) to pain reduction (Secaide), quality of life improvement (BD4QoL) and irritable bowel syndrome management (Zemedy). In the other 15 studies, commercialisation had not been reported (*n* = 7, 25%) or was not clearly reported (*n* = 9, 32%). None of the commercialised CAs included in the studies followed all four translation research stages (development, feasibility, effectiveness and implementation). Most commercialised CAs have conducted at least one effectiveness trial, but only one (PARO) conducted a feasibility study before the effectiveness trial.

### 3.5 Outcomes

Twenty of the included studies reported outcomes. Our primary outcomes of interest were pain, physical function and evaluation of CAs. There were three pain‐related outcomes: pain intensity, pain interference and pain communication. Physical function was reported in five studies. Evaluation of the CAs was reported across two domains: engagement (*n* = 9) and usefulness (*n* = 5). Psychological outcomes were reported in six studies. Outcomes related to depression (*n* = 4), anxiety (*n* = 3) and stress (*n* = 1) were reported. Other qualitative self‐reported outcomes were also noted, such as distress in practical problems among patients (*n* = 1) and effort, mental demand and frustration among health professionals (*n* = 1). Appendix S2 shows outcome measures of interest stratified by the evaluation stage.

### 3.6 Development

One study reported data from the development phase of a computer‐aided learning system. The study compared the accuracy of different machine learning models to predict five diagnoses related to abdominal pain. The goal of the development stage was to utilise the most appropriate model to generate personalised feedback for students about specific patient information requested and their diagnostic hypotheses.

### 3.7 Feasibility

All eight feasibility studies measured users' experiences with CAs, including engagement (*n* = 5), gestures participants used with the CA to express pain (*n* = 1), empathy and perceived intelligence of the CA using questionnaires (*n* = 1) and general experiences with the CA through interviews with users (*n* = 3). Three feasibility studies compared the effects on pain‐related outcomes (*n* = 3), physical function (*n* = 2) and psychological factors (*n* = 2) between baseline and post‐intervention. The results are heterogeneous.

### 3.8 Effectiveness

#### 3.8.1 Pain‐Related Outcomes

Six RCTs reported results on pain intensity. All six studies reported differences between groups. Four studies that reported differences between groups found significant improvements in pain intensity. The other two studies found no significant differences.

Two RCTs reported results on pain interference. Both studies reported differences between groups, with no significant group differences.

One RCT reported results on pain communication, indicating a difference between groups. Participants in the intervention group who used the CA to report their pain described, on average, one additional item about their experience (i.e., expressed more pain‐related content) than the control group (i.e., people who viewed a videotape with or without a practitioner pain coach in the videotape).

#### 3.8.2 Physical Function

Two prospective cohort studies reported results on physical function. Both studies reported no significant differences within groups.

#### 3.8.3 Psychological Outcomes

Four RCTs reported results on psychological outcomes.

Two studies measured outcomes related to depression. One study reported no difference between groups. Another study showed that CA improved depressive symptoms in the completer analysis. However, the benefits ceased to be statistically significant when the intention‐to‐treat analysis was included.

One RCT that reported anxiety outcomes showed no differences between groups. One study measured distress‐related outcomes (0 to 10 scale, 10 being the worst on practical everyday issues) with no significant differences between groups. Another RCT reported outcomes for stress, showing statistically significant differences between groups.

Additionally, one RCT focused on psychological outcomes among healthcare professionals treating patients exposed to CA, finding significant changes within groups in feelings of frustration, mental demand and effort before and after these interventions.

### 3.9 Risk of Bias of RCTs

**FIGURE 4:**
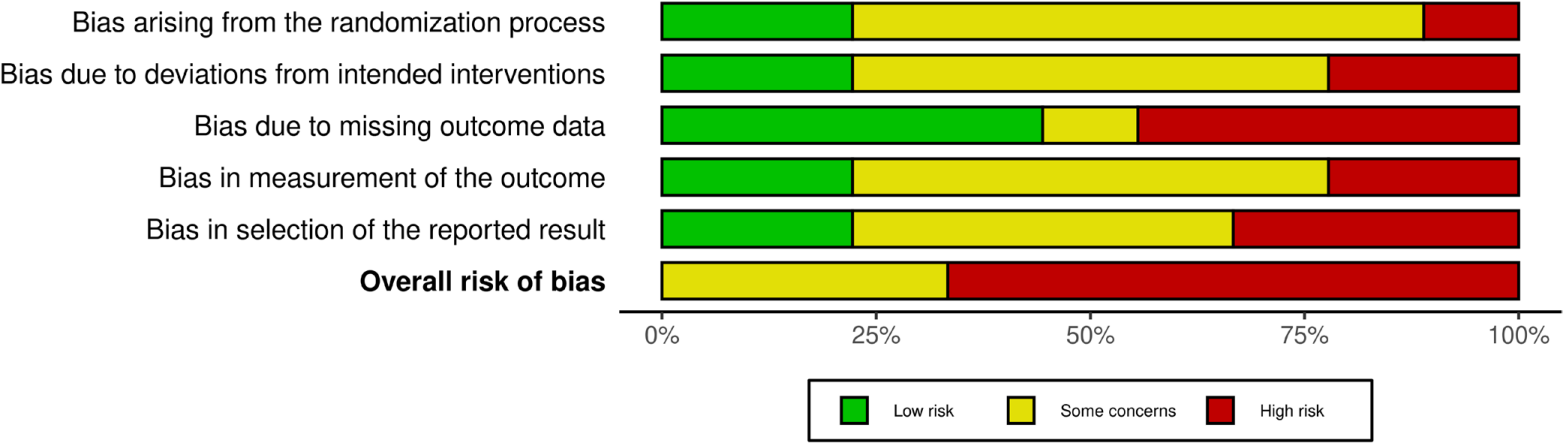
Risk‐of‐bias summary of the included RCTs in the scoping review.

Of the 18 RCTs included in our scoping review, the risk of bias assessment was not possible for nine studies: protocols (*n* = 3), trial registries (*n* = 4) or abstracts (*n* = 2). We assessed nine RCTs for risk of bias (Appendix S2) and provided a summary for the risk‐of‐bias assessment (RoB 2) in Figure 4.

## 4 Discussion

Our scoping review investigated the applications and effectiveness of CAs for supporting pain management in adults. CAs differ from other digital health interventions by providing an interactive, adaptive and often personalised user experience. While traditional digital interventions, such as mobile apps and web‐based programmes, typically rely on static content delivery, CAs engage users through real‐time dialogue, enabling dynamic responses based on user input (Laranjo et al. 2018). We showed that the interest in CAs for pain management is relatively new; most published and unpublished studies (90%) have been reported between 2019 and 2024. Most CAs for pain management were designed to capture health information, facilitate adherence to self‐management and offer emotional/psychological support for patients. CAs have been studied mainly in people with chronic pain‐related conditions such as cancer pain, musculoskeletal conditions, dementia, irritable bowel syndrome, peripheral artery disease and abdominal conditions when patient‐centred assessments, information, advice and self‐management approaches play a critical role in the treatment (Carville et al. 2021).

Among these conditions, dementia is particularly relevant given the high prevalence of pain in this population and the challenges associated with its assessment and management (e.g., cognitive decline and communication barriers). Nearly half of individuals diagnosed with dementia have at least one documented pain‐related condition (Lin et al. 2018) and co‐occurring chronic pain significantly exacerbates self‐care limitations and disability (Haque et al. 2025). Digital health solutions, such as CAs, might assist caregivers in tracking cognitive and symptom changes, monitoring daily routines and providing structured reminders for essential tasks, such as taking medication, maintaining hydration or engaging in social activities. Given the growing interest in AI‐driven tools for healthcare, further research is warranted to explore how CAs can effectively support underserved populations (Lott et al. 2024).

Most of the research on CAs for pain management intended to evaluate the effectiveness of the intervention (18/28, 64%). However, only half of the RCTs have been published (i.e., other studies were described in protocols and trial registries without results). Of 14 unique technologies identified in the 18 RCTs, only one reported being investigated in a feasibility study to guide the main trial (Pu et al. 2019), and none provided sufficient details or references on how the CA was developed for the trial. This is concerning because the lack of development (e.g., reliability of the tool) and feasibility studies (e.g., usability, usefulness, acceptability, desirability of the tool in a particular context) before conducting RCTs may interfere with the recruitment process, follow‐up rates, effectiveness and adherence to the intervention (Martinengo et al. 2023).

Each translational stage (i.e., development, feasibility, effectiveness and implementation) can provide insightful details about the technological aspects of the CAs, users' experiences or effectiveness of the intervention and support commercialisation and clinical application (Wang et al. 2023). Most commercialised CAs (4/5, 80%) were evaluated through RCTs specific to pain management. The CA without an RCT for pain conditions, Wysa, has been investigated in several feasibility studies and has been incorporated in an ongoing RCT for mental health (NCT05943418 2023). Regulatory agencies, such as the Food and Drug Administration (FDA) in the USA (The Food and Drug Administration [FDA] 2023) and the Therapeutic Goods Administration (TGA) in Australia (The Therapeutic Goods Administration [TGA] 2024), regulate the commercialisation process of digital technologies for medical and well‐being purposes. The regulation for medical purposes has more stringent regulatory controls, including the tool's reliability and transparency, efficacy and safety assessments, which implies that most CAs would not be approved if the primary intention is to change patients' health outcomes.

Most CAs for pain management required users to use text messages as the primary mode of communication (i.e., text‐based input interface), whereas some used voice, and a few used gesture and movement input modes. Stand‐alone text‐message interaction is commonly used to provide health education and psychological support and capture patient health information. Multimodal input or output interfaces (e.g., text, voice or visual) are commonly used to provide education or emotional support to patients. Gesture/movement interfaces are combined with virtual reality, avatars or robots to provide emotional support to patients.

CAs for pain management commonly use AI, and some use NLP or machine learning technologies. Most technologies are characterised by a closed knowledge domain (i.e., questions have a predefined answer), constrained (i.e., pre‐programmed options) and interpersonal (i.e., without much intimate connection with users). This is expected to change in the years to come due to the popularity of Open AI technology, which allows users to interact with the CA on various topics without being limited to a specific set of answers. In our scoping review, we only identified one recent RCT protocol, published in 2023 (Blasco et al. 2023), using an open knowledge domain, unconstrained and intrapersonal user interaction. After knee replacement, this text‐based chatbot will be given to patients to increase adherence to home physiotherapy.

Most studies did not explicitly report the characteristics of the CAs. Our research team occasionally used external sources to identify the technology and service provision and determine if the CA had been commercialised. Poor reporting of interventions is common in medical research (McGrath et al. 2024) but may be improved through reporting guidelines. In 2020, two reporting guideline extensions for interventions involving artificial intelligence were published: The SPIRIT‐AI (Standard Protocol Items: Recommendations for Interventional Trials‐Artificial Intelligence) for protocols (Liu et al. 2020) and the CONSORT‐AI (Consolidated Standards of Reporting Trials‐Artificial Intelligence) for clinical trials (Rivera et al. 2020). The CHART (Chatbot Assessment Reporting Tool) reporting guideline has been developed to improve the reporting standards of chatbot assessment studies (Collaborative 2024). As these guidelines are recent, the studies evaluated in this scoping review did not have these tools available to guide the reporting of their findings; however, we recommend that future research adopt reporting guidelines to improve the quality of evidence.

The effects of CAs on patient outcomes are still very uncertain. The RCTs were judged as having a high or some risk of bias, and we could not pool the effects of CAs primarily due to the population's heterogeneity. Overall, CAs using psychoeducation and emotional support appeared not to provide significant benefits in reducing pain interference and function, with mixed results in reducing pain intensity and psychological factors related to chronic pain conditions. Although CAs can be used to improve evidence‐based practice and facilitate shared decision‐making, the current studies in pain management do not explore these applications. Our finding is consistent with a recent Cochrane systematic review that did not find CAs used as decision aids in 209 RCTs (Stacey et al. 2024). Decision‐making tools to faciliate evidence‐based practice commonly use paper‐based or web‐based platforms.

Recently, the potential of using CAs has been increasingly discussed in healthcare, including pain management (Martinengo et al. 2023; Rossettini et al. 2023). For patients, based on input collected in standardised questionnaires or conversational manner, CAs can provide tailored information on rehabilitation exercises, pain management strategies and self‐care techniques, remind patients to use effective communication strategies to self‐manage their conditions, and translate medical terminologies into patient‐friendly language to close the gap between evidence and practice. Chatbots can assist clinicians in clinical reasoning, diagnosis and administrative tasks, such as summarising patients' reports and scheduling and delivering shared decision‐making. Besides their potential applications, CAs also pose risks such as privacy concerns, misinformation, lack of personalisation, overreliance on technology, dataset bias and errors. These challenges can be partially mitigated through transparent development, feasibility and effectiveness studies and regulatory assessments before commercialisation.

### 4.1 Limitations

This study has some limitations that should be noted. Despite combining different study designs, conducting a meta‐analysis of the included RCTs was impossible due to the high heterogeneity among their populations, interventions, comparisons and individual analyses. After screening initial records, we clarified in our protocol registration that the GRADE assessment would be conducted only if meta‐analyses were performed. Although we use a broad definition for CAs based on previous studies, we acknowledge that CAs may be defined differently (Martinengo et al. 2023), which could have potentially affected the inclusion of studies in our scoping review. Another limitation of this review is that although we did not restrict the inclusion of studies based on language, our literature search was conducted exclusively in English‐language databases, which may have inadvertently limited our review to predominantly English‐language studies.

## 5 Conclusion

This scoping review found the use of CAs to support pain management is relatively new, with most studies published in the last 5 years. Currently, CAs are used in pain management to provide education, emotional and psychological support, and to capture health data in a more natural and interactive way to guide treatment and facilitate decision‐making, particularly for chronic pain conditions. The effectiveness of CAs in changing pain‐related outcomes remains highly uncertain. Meta‐analyses cannot be conducted due to a lack of trials examining similar conditions. In addition, we found that there were more RCTs than preliminary studies, such as qualitative studies, reliability assessments and feasibility trials, conducted before the trials. Future RCTs should be informed by preliminary findings on users' experiences, the reliability of CAs and feasibility studies.

## Author Contributions

R.R.N.R., F.Y. and J.H.M. conceived and designed the review. H.B., F.Y. and R.R.N.R. generated the search strategy and searched the databases. F.L.S., H.B., F.Y., J.L., Y.L.G., M.D.J., R.Z.‐P. and R.R.N.R. conducted the screening and data extraction. F.L.S., H.B., F.Y. and R.R.N.R. conducted the first draft. All authors contributed to subsequent edits and approved the manuscript.

## Conflicts of Interest

R.R.N.R. provided consultancy on pain education content for a digital health company not related to conversational agents. The other authors have no conflicts of interest to declare.

## Supporting information


Appendix S1

Appendix S2

## Data Availability

The data that support the findings of this study are available from the corresponding author upon reasonable request.
