# Supplementary material for: Conversational Agents to Support Pain Management: A Scoping Review
Source: Eur J Pain. 2025 Apr 1;29(5):e70016. doi: 10.1002/ejp.70016 (PMC11962237; doi:10.1002/ejp.70016)
Supplement: Supplementary file 1 — Appendix S1 [file EJP-29-0-s002.docx]

| # | **Search** | **Selection** | **Number of records** |
| --- | --- | --- | --- |
| **Section** | **Conversational agents** | |  |
|  | Ai agent | tiab | 33 |
|  | Ai agents | tiab | 43 |
| 1 | Ai agent* | tiab | 73 |
|  | Artificial agent | tiab | 94 |
|  | Artificial agents | tiab | 246 |
| 2 | Artificial agent* | tiab | 309 |
|  | Artificial intelligence chatbot | tiab | 30 |
|  | Artificial intelligence chatbots | tiab | 27 |
| 3 | Artificial intelligence chatbot* | tiab | 55 |
|  | Assistance technology | tiab | 48 |
|  | Assistance technologies | tiab | 23 |
| 4 | Assistance technolog* | tiab | 68 |
|  | Chat bot | tiab | 16 |
| 5 | Chat bot* | tiab | 26 |
|  | Chatbot | tiab | 758 |
|  | Chatbots | tiab | 528 |
| 6 | Chatbot* | tiab | 975 |
| 7 | Chatterbot* | tiab | 1 |
|  | Conversational agent | tiab | 318 |
|  | Conversational agents | tiab | 304 |
| 8 | Conversational agent* | tiab | 479 |
| 9 | Conversational AI* | tiab | 34 |
|  | Conversational assistant | tiab | 5 |
|  | Conversational assistants | tiab | 7 |
| 10 | Conversational assistant* | tiab | 10 |
| 11 | Conversational bot | tiab | 37 |
| 12 | Conversational bots | tiab | 22 |
|  | Conversational bot* | tiab | Asterisk was ignored, must use 4 or more characters for wildcard search |
|  | Conversational interface | tiab | 14 |
|  | Conversational interfaces | tiab | 9 |
| 13 | Conversational interface* | tiab | 22 |
|  | Conversational system | tiab | 12 |
|  | Conversational systems | tiab | 14 |
| 14 | Conversational system* | tiab | 24 |
|  | Dialog system | tiab | 27 |
|  | Dialog systems | tiab | 27 |
| 15 | Dialog system* | tiab | 48 |
| 16 | Dialogue agent* | tiab | 8 |
|  | Dialogue system | tiab | 76 |
|  | Dialogue systems | tiab | 66 |
| 17 | Dialogue system* | tiab | 122 |
|  | Digital assistant | tiab | 639 |
|  | Digital assistants | tiab | 557 |
| 18 | Digital assistant* | tiab | 1106 |
| 19 | Infobot* | tiab | 2 |
|  | Intelligent agent | tiab | 102 |
|  | Intelligent agents | tiab | 175 |
| 20 | Intelligent agent* | tiab | 263 |
| 21 | Intelligent conversational assistant* | tiab | 4 |
|  | Interactive agent | tiab | 39 |
|  | Interactive agents | tiab | 83 |
| 22 | Interactive agent* | tiab | 118 |
|  | Relational agent | tiab | 35 |
|  | Relational agents | tiab | 19 |
| 23 | Relational agent* | tiab | 43 |
|  | Social robot | tiab | 284 |
|  | Social robots | tiab | 466 |
| 24 | Social robot* | tiab | 696 |
| 25 | Virtual advisor* | tiab | 18 |
|  | Virtual agent | tiab | 141 |
|  | Virtual agents | tiab | 148 |
| 26 | Virtual agent* | tiab | 231 |
|  | Virtual assistant | tiab | 108 |
|  | Virtual assistants | tiab | 84 |
| 27 | Virtual assistant* | tiab | 164 |
|  | Virtual avatar | tiab | 91 |
|  | Virtual avatars | tiab | 39 |
| 28 | Virtual avatar* | tiab | 115 |
|  | Virtual coach | tiab | 74 |
|  | Virtual coaches | tiab | 21 |
| 29 | Virtual coach* | tiab | 140 |
| 30 | Virtual conversational agent* | tiab | 6 |
| 31 | embodied conversational agent* | tiab | 86 |
| 32 | avatar* | tiab | 2050 |
| 33 | speech recognition software* | tiab | 94 |
| 34 | voice recognition software* | tiab | 45 |
| 35 | virtual assistan* | tiab | 177 |
| 36 | virtual nurs* | tiab | 78 |
| 37 | virtual patient* | tiab | 1556 |
| 38 | assistance technol* | tiab | 68 |
| 39 | intelligent assistan* | tiab | 70 |
| 40 | digital assistan* | tiab | 1155 |
| 41 | natural language interface* | tiab | 50 |
| 42 | interactive computer agent* | tiab | 17 |
| 43 | computer-assisted instruction* | tiab | 721 |
| 44 | natural language communication* | tiab | 22 |
| 45 | natural language understanding* | tiab | 138 |
| 46 | unconstrained natural language processing* | tiab | 7 |
| 47 | Speech recognition software | mh | 813 |
| 48 | #1 OR #2 OR #3 OR #4 OR #5 OR #6 OR #7 OR #8 OR #9 OR #10 OR #11 OR #12 OR #13 OR #14 OR #15 OR #16 OR #17 OR #18 OR #19 OR #20 OR #21 OR #22 OR #23 OR #24 OR #25 OR #26 OR #27 OR #28 OR #29 OR #30 OR #31 OR #32 OR #33 OR #34 OR #35 OR #36 OR #37 OR #38 OR #39 OR #40 OR #41 OR #42 OR #43 OR #44 OR #45 OR #46 OR #47 | | |
|  | "ai agent*"[Title/Abstract] OR "artificial agent*"[Title/Abstract] OR "artificial intelligence chatbot*"[Title/Abstract] OR "assistance technolog*"[Title/Abstract] OR "chat bot*"[Title/Abstract] OR "chatbot*"[Title/Abstract] OR "chatterbot*"[Title/Abstract] OR "conversational agent*"[Title/Abstract] OR "conversational ai*"[Title/Abstract] OR "conversational assistant*"[Title/Abstract] OR (("conversant"[All Fields] OR "conversants"[All Fields] OR "conversation"[All Fields] OR "conversational"[All Fields] OR "conversations"[All Fields] OR "conversed"[All Fields] OR "conversing"[All Fields]) AND "bot"[Title/Abstract]) OR (("conversant"[All Fields] OR "conversants"[All Fields] OR "conversation"[All Fields] OR "conversational"[All Fields] OR "conversations"[All Fields] OR "conversed"[All Fields] OR "conversing"[All Fields]) AND "bots"[Title/Abstract]) OR "conversational interface*"[Title/Abstract] OR "conversational system*"[Title/Abstract] OR "dialog system*"[Title/Abstract] OR "dialogue agent*"[Title/Abstract] OR "dialogue system*"[Title/Abstract] OR "digital assistant*"[Title/Abstract] OR "infobot*"[Title/Abstract] OR "intelligent agent*"[Title/Abstract] OR (("intelligence"[MeSH Terms] OR "intelligence"[All Fields] OR "intelligences"[All Fields] OR "intelligent"[All Fields] OR "intelligently"[All Fields] OR "intelligibilities"[All Fields] OR "intelligibility"[All Fields] OR "intelligible"[All Fields]) AND "conversational assistant*"[Title/Abstract]) OR "interactive agent*"[Title/Abstract] OR "relational agent*"[Title/Abstract] OR "social robot*"[Title/Abstract] OR "virtual advisor*"[Title/Abstract] OR "virtual agent*"[Title/Abstract] OR "virtual assistant*"[Title/Abstract] OR "virtual avatar*"[Title/Abstract] OR "virtual coach*"[Title/Abstract] OR "virtual conversational agent*"[Title/Abstract] OR "embodied conversational agent*"[Title/Abstract] OR "avatar*"[Title/Abstract] OR "speech recognition software*"[Title/Abstract] OR "voice recognition software*"[Title/Abstract] OR "virtual assistan*"[Title/Abstract] OR "virtual nurs*"[Title/Abstract] OR "virtual patient*"[Title/Abstract] OR "assistance technol*"[Title/Abstract] OR "intelligent assistan*"[Title/Abstract] OR "digital assistan*"[Title/Abstract] OR "natural language interface*"[Title/Abstract] OR (("interact"[All Fields] OR "interactant"[All Fields] OR "interactants"[All Fields] OR "interacted"[All Fields] OR "interacting"[All Fields] OR "interaction"[All Fields] OR "interactional"[All Fields] OR "interactions"[All Fields] OR "interactive"[All Fields] OR "interactively"[All Fields] OR "interactives"[All Fields] OR "interactivities"[All Fields] OR "interactivity"[All Fields] OR "interacts"[All Fields]) AND "computer agent*"[Title/Abstract]) OR "computer assisted instruction*"[Title/Abstract] OR (("natural"[All Fields] OR "naturally"[All Fields] OR "naturals"[All Fields] OR "nature"[MeSH Terms] OR "nature"[All Fields] OR "nature s"[All Fields] OR "natures"[All Fields]) AND "language communication*"[Title/Abstract]) OR "natural language understanding*"[Title/Abstract] OR ("unconstrained"[All Fields] AND "natural language processing*"[Title/Abstract]) OR "speech recognition software"[MeSH Terms] | | |
| **Section** | **Pain** |  |  |
| 49 | Pain Management | mh |  |
| 50 | Pain | mh |  |
| 51 | Chronic Pain | mh |  |
| 52 | #49 OR #50 OR #51 |  |  |
|  | "pain management"[MeSH Terms] OR "pain"[MeSH Terms] OR "chronic pain"[MeSH Terms] | | |
|  |  |  |  |
| **Section** | **Combination** |  |  |
|  | #48 AND #52 |  |  |
|  |  |  |  |
| **Search date** | **Total number** |  |  |
| 1/12/2024 | 104 |  | Added to Covidence |
