# Supplementary material for: Conversational Agents to Support Pain Management: A Scoping Review
Source: Eur J Pain. 2025 Apr 1;29(5):e70016. doi: 10.1002/ejp.70016 (PMC11962237; doi:10.1002/ejp.70016)
Supplement: Supplementary file 2 — Appendix S2 [file EJP-29-0-s001.docx]

**Conversational Agents for Pain Management: A Scoping Review**

Appendix 2 - Data Extraction

Two authors (RRNR and FY) developed and pilot-tested a data extraction form. At least two independent reviewers (HB, RRNR, YG, JL and MJ) independently extracted data from all eligible records. Discrepancies were resolved through discussion with a third reviewer, if necessary. Data were extracted using an Excel data extraction form to obtain the following details:

- Study details (e.g., authors, date, country, objectives, sponsorship source, conflicts of interest)
- Study design (i.e., RCT, case study, case-series, or observational studies, including quantitative and qualitative studies)
- Evaluation stage[1]: development, feasibility, effectiveness, implementation and commercialisation.
- Participants' characteristics (i.e., age, sex, education, race, healthy individuals, clinical population, or health providers), sample size, pain population, and pain duration.
- Characteristics of the intervention: setting (e.g. aged care, community, primary care), recruitment strategy, intervention approach (e.g. psychological, health literacy, informative), intervention target (e.g. weight loss, pain, function, behaviour change), intended co-interventions, length of treatment, and frequency/duration of intervention.
- Characteristics of the conversational agent: name, developer, type (e.g. chatbot, voice assistant), description of chatbot intervention, knowledge domain (open domain: can talk about any subject; closed domain: limited knowledge base)[3], constraint domain (constrained: users can only select from pre-programmed options; unconstrained: users can speak/type freely)[2], service provided (interpersonal: transmits information without much intimate connection to users; intrapersonal: provides companionship or support; interagent: communicates with other CAs or computer systems)[4], goal of interaction (informative: provides stored information; conversational: interacts like a human; task-based: focus on a specific goal)[4], the technology used (AI and machine learning, natural language processing; or generative AI), user interface (text-based; voice-based; visual-based, or multi-modal), commercialisation (whether the conversational agent is commercially available), personalisation (whether the conversational agent can customise responses based on individual user history or preferences), and security and privacy (whether the conversational agent protects the user’s data and ensures privacy). If the study does not describe the characteristics of the technology, researchers will conduct research outside the study to identify the necessary information.
- Outcomes: timepoint, outcome measure, results, conclusion.

**Table 1A.** Reporting of the evaluation stages and commercialisation of each included study

| **Chatbot name** | **First Author, Year** | **Development** | **Feasibility** | **Effectiveness** | **Implementation** | **Commercialisation** |
| --- | --- | --- | --- | --- | --- | --- |
| BD4QoL | Cavalieri, 2023 |  |  | X |  | X |
| Hyrid Ubiquitous Coaching | Kowatsch, 2021 |  |  |  | X |  |
| Northwell Head and Neck Health Chats | Ma, 2021 |  | X |  |  |  |
| Nurse AMIE | Truica, 2022 |  |  | X |  |  |
|  | Schmitz, 2023 |  |  | X |  |  |
| PARO | Demange, 2019 |  |  | X |  | X |
|  | Pu, 2019 |  | X |  |  | X |
|  | Pu, 2020 |  |  | X |  | X |
|  | Pu, 2021 |  |  | X |  | X |
|  | Pu, 2022 |  |  | X |  | X |
|  | Pu, 2023 |  | X |  |  | X |
|  | ACTRN12621001010886; 2021 |  |  | X |  | X |
| Secaide Ver 0.9 | Anan, 2021 |  |  | X |  | X |
| SELMA | Hauser-Urich, 2020 |  |  | X |  |  |
| Wysa | Leo, 2022 |  | X |  |  | X |
|  | Sinha, 2022 |  | X |  |  | X |
|  | Cheng, 2023 |  | X |  |  | X |
| Zemedy | Hunt, 2021 |  |  | X |  | X |
| NR | McDonald, 2011 |  |  | X |  |  |
|  | McDonald, 2012 |  |  | X |  |  |
|  | Khumrin, 2017 | X |  |  |  |  |
|  | Junior, 2020 |  | X |  |  |  |
|  | Silva, 2022 |  |  | X |  |  |
|  | Blasco, 2023 |  |  | X |  |  |
|  | Murali, 2023 |  | X |  |  |  |
|  | NCT06070415 |  |  | X |  |  |
|  | NCT06070441 |  |  | X |  |  |

NR = Not reported

**Table 2A.** Outcome measures stratified by the evaluation stage

| **Evaluation Stage** | **First author, year** | **Outcome Measures of Interest** | **Results / Conclusions** |
| --- | --- | --- | --- |
| Development | Khumrin, 2017 | Performance of the CA that used machine learning (interpretation of diagnoses of abdominal pain) | E-learning has a role in the development of medical students’ diagnostic reasoning skills (overall accuracy = 85.1%). |
| Feasibility | Cheng, 2023 | Physical Function | No significant difference from baseline M = 38.6, SD = 7.2) to 1 month post intervention (M = 39.9, SD = 5.6; *P*=.26). |
|  |  | User’s experiences (engagement) | Total interactions divided by the number of participants who completed onboarding provides an engagement rate of 68%. |
|  |  | Pain Interference | Significant improvement from baseline (M = 64.2, SD = 7.2) to 1 month post intervention (M = 60.7, SD = 6.9, *P*=.002). |
|  |  | Anxiety | Significant improvement within group from baseline to one month follow-up (MD –3.2, –4.3 to –2.0). |
|  |  | Depression | No significant difference within groups from baseline to one month follow-up (MD –1.3, –2.9 to 0.3). |
|  | Junior, 2020 | User’s experiences (engagement) | Adherence was considered satisfactory with a 73% engagement rate for questions about oral medication use. |
|  | Leo, 2022 | User’s experiences (engagement) | The overall rate of engagement was 72%. Retention rate was 84%. |
|  |  | Physical Function | No significant difference within groups from baseline (M = 35.9, SD = 6.6) to 2-month follow up (M = 39.5, SD = 6.7; *P*=.99). |
|  |  | Pain Interference | No significant difference within groups from baseline (M = 65.2, SD = 6.5) to 2-month follow up (M = 62.1, SD = 7; *P*=.26). |
|  |  | Depression | Difference within groups from baseline (M = 58.1, SD = 7.3) to the 2-month follow-up (M = 54.7, SD = 78.7). Significance was not provided. |
|  |  | Anxiety | Difference within groups from baseline (M = 62.2, SD = 5.9) to the 2-month follow-up (M = 58, SD = 7.8). Significance was not provided. |
|  | Ma, 2021 | User’s experiences (engagement) | The total engagement during the study period was 71%. |
|  |  | Pain intensity scores between patients and clinicians. | 31% agreement (pain was underestimated by clinicians in 40%, but the difference was not significant). |
|  | Murali, 2023 | Gestures to express pain during interaction with the agent. | Interactions were significantly longer in empathetic condition (empathetic feedback that repeated what the users said about their pain) than report only condition. |
|  |  | User’s experiences (empathy, perceived intelligence, interviews) | Significant difference between the intervention group (empathetic feedback that repeated what the users said about their pain) and control group on likeability (Z = -2.37, *P* = .02) and perceived intelligence (Z = -2.20, *P*= .03).  Interviews with participants showed that empathetic conditions are more empathetic. For example, a participant from the report only condition said ‘She [the CA] understood it [my pain], but she didn’t really repeat it back to me." |
|  | Pu, 2019 | User’s experiences (interviews with users) | Interviews with residents with dementia showed that they expressed positive attitudes towards the use of the CA and acknowledged the therapeutic benefits on mood improvement and relaxation for pain relief. |
|  | Pu, 2023 | User’s experiences (interviews with users, observers, such as family and carers) | Interviews with residents with dementia, family members, and formal carers found the CA was helpful in reducing pain and the associated behavioural symptoms, and in the assessment of pain. |
|  | Sinha, 2022 | User’s experiences (engagement) | Users engaged in a mean of 4.0 (SD 0.9) sessions per week and a mean of 33.3 (SD 42) total sessions during the 8-week study period. The retention rate at 1 month was 70%. |
| Effectiveness | Anan, 2021 | Pain Intensity | Significant difference between the intervention group (M = 3.0, SD = 1.1) and control group (M = 4.0, SD = 0.8) at 12-week follow up (P<.001). |
|  |  | User’s experiences (engagement) | Adherence rate was 92%. |
|  | Demange, 2019 | Pain Intensity | Behavioural manifestations of pain were significantly lower in the CA-mediated intervention period compared to the control period (p<.0001). |
|  |  | Effort | Health professionals reported significantly less effort within groups (p<.05) |
|  |  | Frustration | Health professionals reported significantly less frustration within groups (p<.01) |
|  |  | Mental demand | Health professionals reported significantly less mental demand within groups (p<.01) |
|  | Hauser-Urich, 2020 | Pain Intensity | Significant group differences from baseline to post intervention, with the intervention group showing lower pain intensity (t37=–2.8, P=.009). |
|  |  | Pain Interference | No significant group differences from baseline to post intervention (t60=0.42, *P*=.68). |
|  |  | User’s experiences (engagement) | Adherence rate was 71%. |
|  | Hunt, 2021 | Pain Intensity | Significant group differences from baseline (M = 36.76, SD = 12.77) to 8-week follow up (M = 27.56, SD = 10.12), with the intervention group showing lower pain intensity (F1,79=20.12, *P*<.001) |
|  |  | Anxiety | No statistical difference between groups (F1,79=1.84, P=.18, d=0.41). |
|  |  | Depression | Significant difference between groups from baseline (M = 8.32, SD = 5.29) to 8-week follow up (M = 5.78, SD = 4.20), with the intervention group showing lower depression scores measured by PHQ-9 (F1,79=10.5, P=.002, d=1.07) and DASS Depression (F1,79=6.03, P=.02, d=0.83) in the completer analyses. These results were nonsignificant in the intention-to-treat analysis using multiple imputations. |
|  |  | Stress | Significant difference between groups from baseline (M = 17.84, SD = 9.56) to 8-week follow up (M = 12.72, SD = 8.65), with the intervention group showing lower stress scores measured by DASS Stress subscale (F1,79=4.47, P=.04, d=0.65). |
|  | McDonald, 2011 | Pain Communication | Intervention group (interactive virtual coach) described on average one additional item of pain information compared to people who viewed a videotape with or without a practitioner pain coach in the videotape. |
|  | McDonald, 2012 | Pain Intensity | No significant difference between groups at 1-month post intervention. |
|  |  | Pain Interference | No significant between groups at 1-month post intervention. |
|  |  | Depression | No significant between groups at 1-month post intervention. |
|  | Pu, 2020 | User’s experiences (engagement) | Adherence rate was 88.4%. |
|  | Pu, 2021 | Physical Function | No significant differences from baseline to 6 weeks post-intervention (*P*>0.05). |
|  | Schmitz, 2023 | Pain Intensity | No significant group differences from baseline to 3-month follow up (*P*=0.58). |
|  |  | Physical Function | No significant group differences from baseline to 3-month follow up (*P*=0.57). |
|  |  | Distress | No significant differences between groups from baseline to 3-month follow up (P=0.70) |
| Implementation | Kowatsch, 2021 | User’s experiences (engagement, usefulness with patients and physiotherapists) | Adherence rate was 92%.  Patients found the CA to be useful, easy to use, and enjoyable, whilst also expressing an intention to use it again in the future. Physiotherapists perceived an advantage to the use of CA that delivers psychoeducation and motivation messages in the therapy process. |

| **Table 3A.** Risk of bias using Cochrane RoB 2 tool. | | | | | | |
| --- | --- | --- | --- | --- | --- | --- |
| Study | **RoB arising from randomisation process** | **RoB due to deviations from intended interventions** | **RoB due to missing data** | **RoB in measurement of outcome** | **RoB in selection of reported result** | **Overall Risk-of-Bias** |
| Anan, 2021 | High | High | Low | Some | High | High |
| Hauser-Ulrich, 2019 | Some | Some | High | Some | High | High |
| Hunt, 2021 | Some | Some | High | Some | Low | High |
| McDonald, 2011 | Low | Some | Low | Some | High | High |
| McDonald, 2012 | Some | Some | High | High | Some | High |
| Schmitz, 2023 | Low | High | High | High | Some | High |
| Murali, 2023 | Some | Some | Some | Low | Some | Some |
| Pu, 2020 | Some | Low | Low | Some | Some | Some |
| Pu, 2021 | Some | Low | Low | Low | Low | Some |

RoB: Risk of Bias; High: refers to a high risk-of-bias; Some: refers to some risk-of-bias; Low: refers to low risk-of-bias


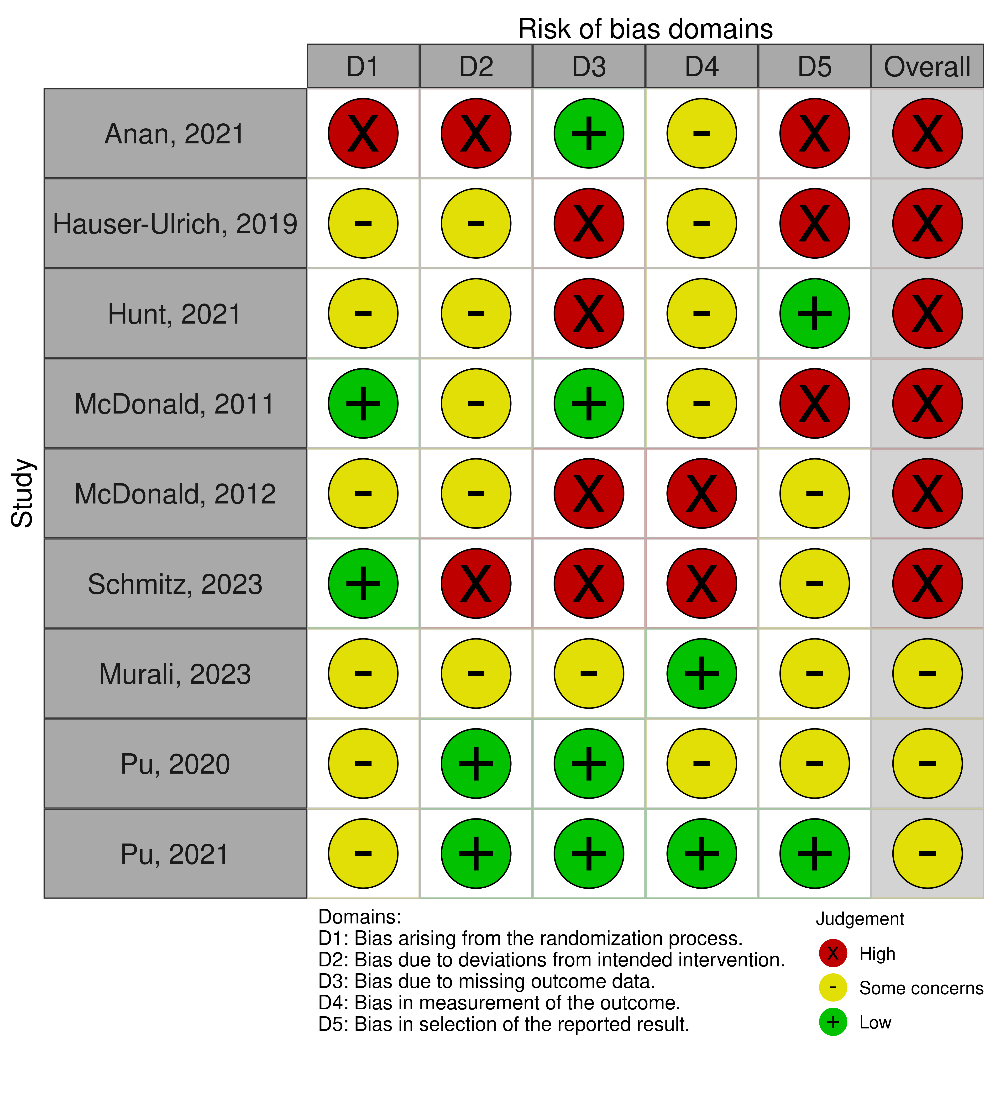
 **Figure 1A.** Colored traffic plot

**References** (Appendix A2)

- [1] Moore GF, Audrey S, Barker M, Bond L, Bonell C, Hardeman W, Moore L, O'Cathain A, Tinati T, Wight D, Baird J. Process evaluation of complex interventions: Medical Research Council guidance. BMJ 2015;350:h1258.
- [2] Oh YJ, Zhang J, Fang ML, Fukuoka Y. A systematic review of artificial intelligence chatbots for promoting physical activity, healthy diet, and weight loss. Int J Behav Nutr Phys Act 2021;18(1):160.
- [3] Powell L, Nizam MZ, Nour R, Zidoun Y, Sleibi R, Kaladhara Warrier S, Al Suwaidi H, Zary N. Conversational agents in health education: Protocol for a scoping review. JMIR Res Protoc 2022;11(4):e31923.
- [4] Xu L, Sanders L, Li K, Chow JCL. Chatbot for Health Care and Oncology Applications Using Artificial Intelligence and Machine Learning: Systematic Review. JMIR Cancer 2021;7(4):e27850.
